# Equity in epidemic response: an action-oriented framework for guiding public health in equitable responses to major infectious disease emergencies

**DOI:** 10.1186/s12939-025-02433-2

**Published:** 2025-03-12

**Authors:** Precious-Junia de-Winton Cummings, Kelly K. Baker, Leah Appell, Marina Del Rios, Daniel J. Diekema, Tricia Kitzmann, Naomi Marroquin, Gus Raymond, Martha L. Carvour

**Affiliations:** 1 Department of Epidemiology, University of Iowa (UI) College of Public Health (CPH), 145 N Riverside Drive, Iowa City, IA 52242 USA; 2 Department of Internal Medicine, University of Iowa Carver College of Medicine (CCOM), 200 Hawkins Drive, Iowa City, IA 52242 USA; 3 Department of Epidemiology and Environmental Health, School of Public Health and Health Professions, University at Buffalo, 168C Farber Hall, Buffalo, NY 14214 USA; 4 Center for Climate Change and Health Equity, 168C Farber Hall, Buffalo, NY 14214 USA; 5 Integrated Multidisciplinary Program of Assertive Community Treatment, UI Health Care, 200 Hawkins Drive, Iowa City, IA 52242 USA; 6 Department of Emergency Medicine, UI Carver College of Medicine (CCOM), 200 Hawkins Drive, Iowa City, IA 52242 USA; 7 Department of Medicine, Maine Medical Center-Maine Health, 110 Free Street, Portland, Maine 04101 USA; 8 Department of Community and Behavioral Health, UI CPH, 145 N Riverside Drive, Iowa City, IA 52242 USA; 9 Proteus, Incorporated, 1121 Center St. Suite 16, Des Moines, IA 50309 USA; 10 Storm Lake Community School District, 419 Lake Avenue, Storm Lake, IA 50588 USA

**Keywords:** Health equity, Infectious disease, Framework, Epidemic response, Pandemic response

## Abstract

**Background:**

A rapid and equitable response is paramount to mitigating the spread and impact of an infectious disease public health emergency. Unfortunately, public health responses often integrate equity as a secondary component rather than a foundational one—a decision that can result in disproportionate effects of the epidemic on vulnerable populations and that may further fuel or worsen the ongoing health emergency. This paper introduces a framework grounded in health equity principles to guide the design and implementation of response efforts during infectious disease emergencies.

**Methods:**

The Equity in Epidemic Response framework was developed by critically appraising and synthesizing several established models into an integrated framework, with active engagement from health professionals specializing in epidemiology, medicine, global health, mental health, community health, and health policy.

**Results:**

The framework covers six high-impact areas that should be addressed during an infectious disease public health emergency: *community partnerships and engagement; communication*;* social and economic conditions; data systems and methods; health infrastructure and supply chains for preventives*,* therapeutics*,* and diagnostics; and accessibility of outbreak resources and essential health services*. Key priorities and assessment indicators within each area were identified.

**Conclusions:**

Given the increasing threat of emerging and re-emerging infectious diseases, this framework reinforces the need to prioritize equitable approaches in responding to infectious disease public health emergencies to minimize health consequences, particularly among vulnerable populations. This framework is designed as a practical tool for public health professionals to guide major aspects of an epidemic response, ensuring thorough and equitable implementation of response efforts.

**Supplementary information:**

The online version contains supplementary material available at 10.1186/s12939-025-02433-2.

## Background

Following the detection of an infectious disease outbreak, a rapid public health response is required to curb the spread of the disease. However, recent epidemics such as the 2014–2016 Ebola outbreak [1, 2], the 2019 coronavirus disease (COVID-19) pandemic [3–7], and the 2022 Mpox outbreak [8–10] have demonstrated disparities in the epidemic response and reinforced the need for equitable approaches to infectious disease public health emergencies.

Multiple elements hinder effective emergency responses, including disjointed partnerships among key sectors, low levels of community involvement in the planning and implementation of response strategies, and inadequate availability or distribution of essential resources [11, 12]. Although public health strategies often acknowledge the importance of equity, they may fail to implement it as a core aspect of the response effort. Hence, vulnerable populations may be disproportionately affected, and pre-existing health inequalities may be exacerbated, even when response actions are implemented.

Evidence from the 2014–2016 Ebola epidemic revealed that response efforts were hindered by poor communication practices, mistrust in government, and limited health infrastructures [13, 14]. The response to the 2022 Mpox outbreak had similar limitations: Racial minority groups experienced higher infection rates, while a lack of disaggregated data limited a detailed understanding of disparities related to social and demographic factors, such as race/ethnicity and gender identity [9, 10, 15]. Notably, messaging about the disease raised concerns about stigmatizing language, which may have delayed care-seeking [8–10]. Likewise, the COVID-19 pandemic exposed significant gaps in the healthcare system, significantly impacting the response to the outbreak. Risk communication methods often lacked cultural sensitivity and did not account for the varying languages represented in the population [16]. Social distancing measures were difficult to implement in larger households or densely populated areas. The diagnostic testing process was hindered by extended wait times, which led to delays in testing, ultimately affecting the effectiveness of disease surveillance [17]. Stay-at-home measures were challenging for low-income individuals and frontline workers who were required to continue working in person, increasing their risk for infection. Many individuals in occupations without paid sick leave or flexible work schedules opted not to seek health services for preventative (e.g., vaccination) or therapeutic care [18]. Physical accessibility to vaccination centers was challenging for those in underserved areas or those lacking transportation access [19, 20].

Achieving equitable policies during disease emergencies requires addressing systemic barriers and embedding health equity considerations into all public health policies, processes, and systems from their inception [21]. This involves accounting for social determinants of health, such as income, housing, and access to healthcare, to ensure that interventions do not inadvertently exacerbate existing inequities [21]. A 2021 CDC report on the impact of the COVID-19 pandemic underscored the importance of collecting complete demographic data to guide prevention efforts for underrepresented populations, fostering community outreach and partnerships, and training public health workers from diverse backgrounds to promote health equity [21]. Improving vaccine manufacturing capacity in low- and middle-income countries is another critical step toward equity. Organizations like the Coalition for Epidemic Preparedness Innovations highlight the importance of geographically diversifying vaccine production to promote regional self-sufficiency and equitable distribution of resources [22]. Additionally, Glover et al. propose a framework to address equity harms in pandemic policies, emphasizing the need to consider structural inequities and mitigate unintended consequences, such as the impact of lockdowns on food insecurity, mental health, and stigma [23]. Similarly, Watts et al. emphasize the need for engaging and educating communities about the purpose of public health laws to foster trust, promote equity, and build resilience [24]. They argue that involving community members in developing and implementing public health measures enhances adherence and improves outcomes during health emergencies [24].

In light of the persistent inequities exposed during past emergencies and the ongoing risk posed by emerging and re-emerging infectious diseases, there is an urgent need for a paradigm shift in public health responses to major infectious disease emergencies. Health equity should be a fundamental element of our epidemic response rather than merely an added component of current models. The critical question is: ***How can public health professionals and health systems respond to pandemics and epidemics fairly and inclusively***? With this question in mind, we sought to develop a practical, theoretically grounded tool to guide equitable responses during major disease emergencies. Existing tools [e.g., the World Health Organization’s International Health Regulations Joint External Evaluation (JEE), Global Health Security Index (GHSI)] primarily focus on technical capacities at the national level while often overlooking systemic inequities, such as disparities in healthcare access and socioeconomic variation within and between populations that may influence effective responses [25–29]. While these tools provide valuable guidance, their broad recommendations lack the actionable specificity needed to effectively address equity-related challenges in public health responses, particularly at local and subnational levels where disparities are often most pronounced.

Addressing these gaps requires tools that incorporate equity-focused metrics in major components of epidemic response, emphasizing the lived experiences of vulnerable populations and the critical importance of community-centered approaches. Here, we examine several existing models with this question in mind and propose an action-oriented conceptual framework rooted in the structural underpinnings of health equity promotion. The resulting framework emphasizes the importance of establishing clear, structural objectives in the epidemic response and fostering inclusive and bidirectional collaboration across sectors. It complements existing tools by providing a robust, equity-focused foundation across several domains of response measures, enhancing their applicability to specific response actions, and addressing gaps in their current approaches to equity. By embedding equity as a core principle, this framework serves as a valuable addition to infectious disease response efforts, strengthening public health decision-making and reducing the burden of disease outcomes. In this paper, we outline the methodology used to develop the Equity in Epidemic Response (EER) framework, describe its components, and provide an assessment tool to enhance the capacity of public health professionals to iteratively gauge and revise a response strategy.

## Methods

### Overview of methods

**Fig. 1:**
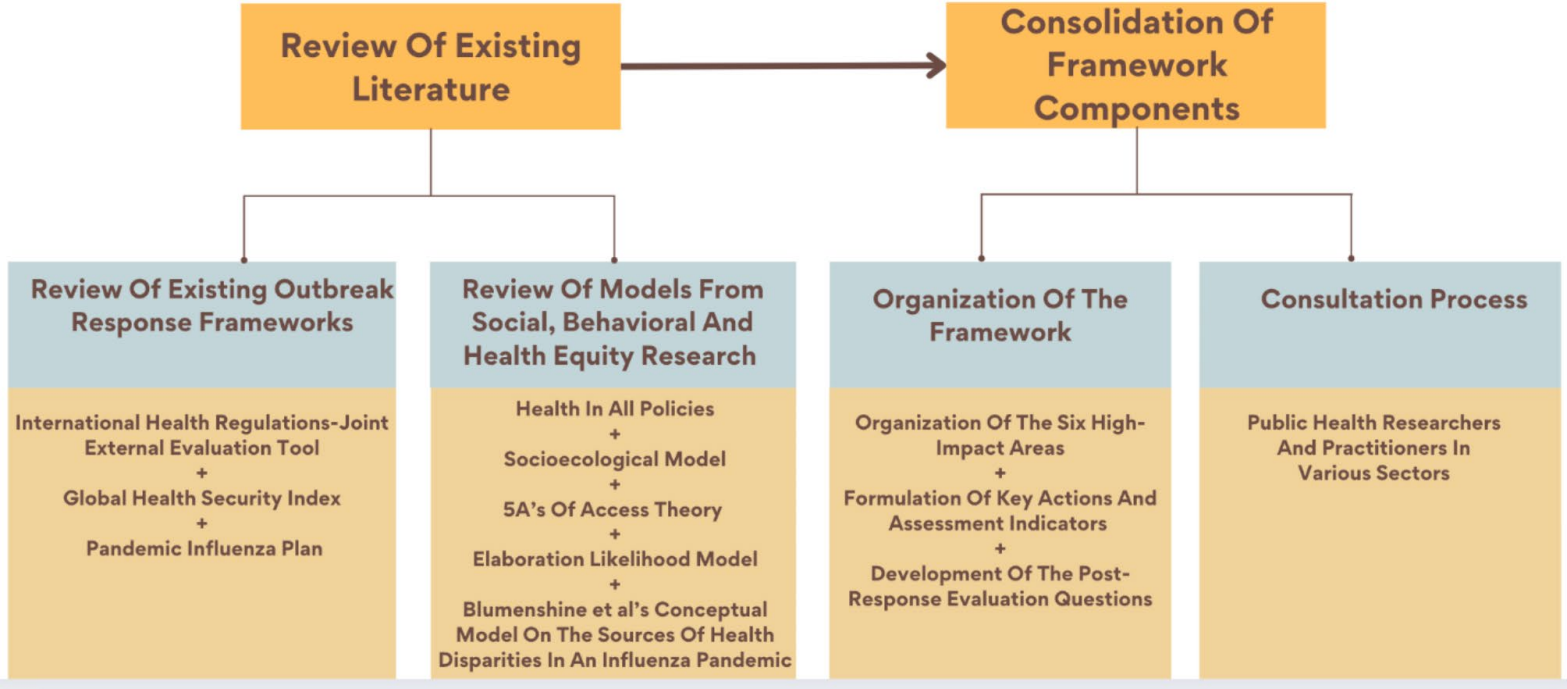
Framework development process

The Equity in Epidemic Response (EER) framework was developed by synthesizing various established frameworks with critical input from public health experts representing diverse professional sectors. First, PJDWC reviewed existing epidemic and pandemic response frameworks to identify their strengths and any health equity-related gaps in their priorities. Second, PJDWC drew upon other social, behavioral, and health equity research models to formulate the framework’s core components. Thereafter, through iterative discussions and synthesis of findings from these reviews with MLC and KKB, PJDWC identified several actionable priorities; and corresponding assessment indicators for each high-impact area were devised. To improve the framework for public health use, six health professionals across different sectors (LA, MDR, DJD, TK, NM, and GR) provided feedback representing their professional experiences, resulting in modifications to the framework. Detailed information about the development process is provided in two broad steps below (Fig. 1).

## Review of existing literature

### Appraisal of existing epidemic/pandemic tools and identifying relevant health models

In the initial phase of formulating this framework, our approach centered on a targeted literature review to address gaps in existing epidemic/pandemic tools and incorporate health models to create a more comprehensive approach to epidemic response. The review process was designed to answer the following key questions: (1) How do existing epidemic/pandemic response tools address health equity, and what are the gaps in their coverage? (2) How can social, behavioral, and health equity research inform the development of a more comprehensive and equity-grounded epidemic response framework?

Our review process was selective, focusing on a few well-known epidemic/pandemic response tools and relevant health theories and models. We examined tools developed by global health organizations and national public health agencies. In addition, we explored health models and theories that promote equity, collaboration, and community involvement, including frameworks such as the Socioecological Model and the 5As of Access theory. A detailed description of the review process is provided in the following two sections.

### Review of existing preparedness and response frameworks

We first examined well-known, existing epidemic/pandemic response frameworks and evaluation tools to identify health-equity-related gaps that can be strengthened by the EER framework. Some of the most widely utilized frameworks over the years include the JEE, the GHSI, and the United States Department of Health and Human Services Pandemic Influenza Plan (PIP). The recent edition of the JEE, which was implemented in 2005, is a framework that outlines the roles and responsibilities of member states in preventing, detecting, and responding to public health emergencies of international concern [25]. The GHSI comprehensively assesses a country’s capability to prevent, detect, and respond to biological threats [28, 29]. The 2017 PIP includes recommendations for influenza outbreaks, such as vaccine development and distribution, laboratory testing, and community mitigation strategies [30].

Although these frameworks have effectively guided many elements of prior epidemic response efforts, they do not sufficiently consider health equity as a fundamental aspect of the response. For instance, the JEE framework primarily focuses on national-level preparedness and response, which limits its consideration of the social and economic causes of health inequities among different populations within a country [25]. The pandemic accentuated the extent to which inequities within and between countries affected the distribution and accessibility of pharmaceutical countermeasures [3, 5, 6]. Recognizing these gaps, the World Health Assembly recommended adjustments to the JEE in December 2021, advocating for a whole-of-society approach that places greater emphasis on health equity [31]. While the latest version of the JEE includes equity metrics, these mainly focus on gender equity during health emergencies. Although this is important, this limited perspective overlooks other essential dimensions of equity, such as racial, socioeconomic, and geographic disparities, which greatly impact health outcomes during crises. As a result, the tool fails to address the wider systemic inequities that disproportionately affect marginalized populations, restricting its ability to guide comprehensive and inclusive public health responses. Additionally, the tool fails to adequately address structural barriers, particularly those that impact the utilization of health services during public health emergencies.

Although the 2019 GHSI index correctly predicted that the world was unprepared for a pandemic, higher scores, which reflected stronger preparedness and response abilities for specific countries, did not necessarily result in positive outcomes during the pandemic. Countries with the highest GHSI scores, such as the United States and United Kingdom, reported more cases and deaths than countries with lower scores [28, 29]. A 2020 study found a positive correlation between national cumulative death rates from COVID-19 and GHSI scores (*r* = 0.35, *P* < 0.001). Although it is challenging to compare morbidity and mortality rates between countries due to differences in testing patterns or case definitions, it is evident that there are significant areas for improvement with the GHSI tool in assessing response capacities [27]. However, some researchers have argued that these tools are not intended to predict outcomes but rather to identify gaps in capacities for an effective response [32]. Like the JEE tool, the 2019 GHSI does not consider health inequalities within countries. The response component of the tool also overlooks social and economic measures to address housing and financial instability, as well as community involvement in response capacities. Effective epidemic and pandemic responses require collaboration with communities, as seen during the COVID-19 pandemic, to encompass diverse perspectives in response planning [33, 34]. The 2021 edition of the GHSI index now incorporates the influence of social and political factors in facilitating effective responses to significant disease events [31]. New measures were added to address misinformation, the availability of health surveillance data, non-pharmaceutical intervention planning, laboratory strength, paid medical leave for health workers, and government effectiveness [31]. However, concerns remain regarding its comprehensiveness, as the index places significant emphasis on factors such as trust and the politicization of public health—which are undoubtedly relevant—but provides less focus on addressing structural and logistical inequities that critically impact health outcomes.

The PIP differs from the JEE and GHSI in considering the federal, state, and local capacities of the United States in pandemic response. While the plan emphasizes plain language communication, it falls short in adequately addressing cultural and linguistic differences, as well as in combating stigma and misinformation, which are critical for fostering trust and adherence. Beyond its focus on communication strategies and the implementation of non-pharmaceutical interventions, the PIP places minimal emphasis on engaging community stakeholders in planning and decision-making processes. This lack of community involvement could hinder efforts to develop culturally appropriate and effective interventions. Additionally, while the PIP aims to expedite resource accessibility, it lacks clear, actionable strategies to ensure equitable distribution and promote uptake, leaving significant gaps in addressing systemic barriers to resource utilization [28]. Despite the ongoing threats of disease emergencies, there has been no recent update to the PIP.

The changes in these tools reflect a growing recognition of the importance of equity-focused considerations in pandemic and epidemic preparedness and response. However, more targeted and actionable strategies are required to address the structural barriers that frequently impede effective responses. The EER framework builds on this progress by complementing existing tools, focusing on strategies that address systemic inequities and prioritize community-centered solutions.

### Review of social, behavioral, and health equity research models

The EER framework was designed to acknowledge the disparities many populations face in accessing health services and, as such, to emphasize equity, community, and collaboration as foundational aspects of effective epidemic responses. To further develop these aspects of the framework, we reviewed relevant models from other social, behavioral, and health equity research. The Health in All Policies (HiAP) Framework, designed to integrate health equity and sustainability into policy development, guided our framework’s emphasis on cross-sector collaboration and community engagement for effective response efforts during disease emergencies [35]. HiAP’s principles of intersectoral collaboration encourage alignment across various sectors to achieve public health goals, while its emphasis on stakeholder engagement stresses the importance of inclusive decision-making that addresses the needs of diverse populations [35]. These elements reinforce the EER framework’s commitment to fostering holistic, community-driven responses. The Socioecological Model, which addresses the dynamic interrelations between social influences and environmental factors on an individual’s health, guided our approach to addressing the broader community, organizational, and societal influences of the epidemic response [36]. By moving beyond the sole focus of individual behavior change, this framework supports a more comprehensive perspective that allows interventions to target multiple layers of influence, enhancing its impact. Blumenshine et al.’s model on the sources of disparities during a pandemic influenza outbreak was leveraged to integrate a focus on structural barriers in health and their potential impact on access to resources [37]. The model highlights critical factors that contribute to these disparities, emphasizing the role of social determinants of health in shaping vulnerability to infection, access to healthcare, and health outcomes [37]. Thomas and Penchansky’s Access theory further shaped the framework by emphasizing the five critical dimensions of access: Affordability, Availability, Accessibility, Accommodation, and Acceptability [38]. These dimensions describe whether individuals can obtain and utilize healthcare services effectively, highlighting the importance of equitable access to preventive care, diagnostics, treatments, and other essential resources during epidemics [38]. Finally, Petty and Cacioppo’s Elaboration and Likelihood Model (ELM) informed our strategies for effective communication and public health messaging. This model is a theory of persuasion that delineates how individuals process persuasive messages and the factors influencing the effectiveness of these messages [39]. Our framework emphasizes the development of messages that raise awareness, generate positive attitudes, establish social norms, and influence behavior, ensuring that communication strategies are both impactful and inclusive.

Together, these models strengthen the EER framework by integrating theory and evidence-based principles that address structural factors, support community-driven approaches, and foster effective communication, ultimately advancing equitable responses to public health emergencies.

### Consolidation of framework components

#### Organization of the high-impact areas, key actions, and assessment indicators

The EER framework consists of four components: high-impact areas, key actions, planning and implementation assessment indicators, and post-response evaluation indicators. Drawing from our review of the strengths and limitations of existing epidemic/pandemic frameworks and the social, behavioral, and equity literature, we identified six high-impact areas for the EER framework: *community partnerships and engagement; communication; social and economic conditions; data systems and methods; health infrastructure and supply chains for preventives*,* therapeutics*,* and diagnostics; and accessibility of outbreak resources and essential health services.* After identifying the six EER high-impact areas, we sought to identify key actions (Table 1) and assessment indicators (Supplementary Material) to ensure each impact area addresses response capacities effectively and equitably. Finally, we proposed a sequence of steps for integrating the components of the EER framework into a robust foundation for addressing epidemics and pandemics.

**Table 1:** High-impact areas and associated key actions in the Equity in Epidemic Response framework

High-Impact Area	Key Actions
**Community Partnerships and Engagement**	• Engage with community stakeholders to identify needs and address barriers with response strategies. • Implement interventions using local resources. • Practice cultural humility and prioritize building trust in communities.
**Communication**	• Engage community stakeholders in designing and disseminating communication plans and materials. • Develop culturally aware messages to prevent stigma, labeling, or othering of populations. • Adapt messages so that information is readily accessible and comprehensible. • Monitor engagement with health messages.
**Social and Economic Conditions**	• Provide safe and temporary shelter. • Strengthen economic security. • Promote social support services.
**Data Systems and Methods**	• Establish mechanisms for equitable and ethical data collection and sharing with researchers and communities. • Increase representation of marginalized populations in research studies. • Implement or strengthen community-based health surveillance. • Utilize qualitative and quantitative methods to design and analyze research studies.
**Health Infrastructure and Supply Chains for Preventives**,** Therapeutics and Diagnostics**	• Promote partnerships to expand the production of preventives, therapeutics, and diagnostics. • Develop an equitable mechanism for procuring, allocating, and delivering vaccines, therapeutics, and diagnostics. • Strengthen local laboratory capacities.
**Accessibility of Outbreak Resources and Essential Health Services**	• Address barriers that may influence the uptake of public health measures. • Develop sustainable supply and distribution strategies. • Provide training and support for health workers, including community health workers. • Maintain the provision of other essential health services such as care for chronic conditions, substance use disorders, sexually transmitted infections, etc.

#### Consultation process

The expertise of six health professionals was sought to provide valuable feedback on the framework components and their applicability across sectors.

First, three academic collaborators (MDR, DJD, and TK) were given documents containing an executive summary of the framework and the assessment indicators. They provided feedback on the entire framework or specific impact areas relevant to their expertise. These three collaborators work in public health and medicine and have expertise in infectious diseases, epidemiology (including hospital epidemiology), microbiology, emergency medicine, community health, health equity, public health practice, and policy. Virtual meetings were held for collaborators who wanted to share additional insights.

Since this framework places significant emphasis on community engagement, the consultation process also included collaborators who serve vulnerable populations due to factors such as age, occupation, immigration status, and mental health and who held crucial roles in the protection of community health during the COVID-19 pandemic (LA, NM, GR). To allow for in-depth discussions about the context of the EER framework in community settings, community collaborators were consulted through virtual, one-on-one discussion sessions. The sessions were structured around discussions of epidemic and pandemic resources, measures that could be taken to protect local communities, and methods to equitably measure public health responses.

The consultation process played a key role in improving the framework. Insights from academic collaborators helped ensure that the framework reflected the best practices in public health research, while input from community collaborators provided valuable perspectives about the framework’s practical implications and relevance for community involvement in a pandemic/epidemic response. The feedback from the six collaborators allowed for adjustments of key actions and assessment indicators and clarification of key concepts.

## Results

Here, we present the EER framework, an action-oriented framework developed by appraising and synthesizing literature and engaging health professionals in academic and community settings to guide responses to major infectious disease public health emergencies.

EER prioritizes action. Its six high-impact areas are mapped to specific key actions and assessment indicators, ensuring that structural and systemic issues are systematically addressed across all high-impact areas. From community engagement to data-driven decision-making, the framework’s suggested actions aim to reduce structural barriers and advance fairness in resource distribution and uptake. By embedding equity-focused principles into the design and implementation of public health responses, the framework seeks to mitigate disparities and foster equitable health outcomes during public health emergencies.

### Description of high-impact areas

**Fig. 2:**
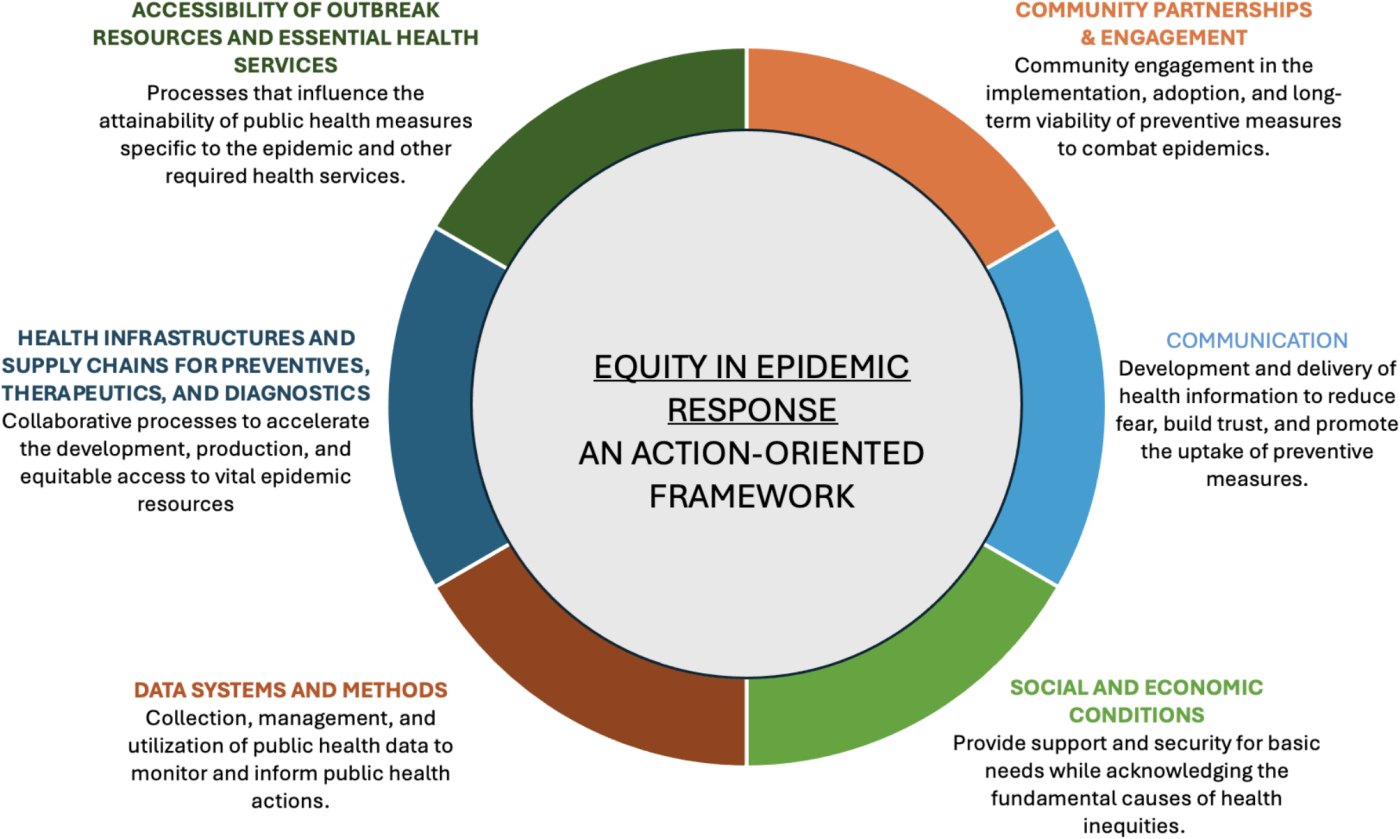
The equity in epidemic response (EER) framework

We define a high-impact area as one that has an immense effect on reducing the burden of an epidemic. The six areas identified emphasize the multi-disciplinary aspect of a pandemic/epidemic response, including but not limited to epidemiology, social and behavioral sciences, health policy, geography, ethics, and clinical and laboratory sciences. We consider each high-impact area a proportion of the whole, i.e., each area should be prioritized and addressed in tandem for an effective response. We acknowledge that the time and resources needed to address each high-impact area may vary. Figure 2 illustrates the six high-impact areas: *community partnerships and engagement; communication; social and economic conditions; data systems and methods; health infrastructure and supply chains for preventives*,* therapeutics*,* and diagnostics; and accessibility of outbreak resources and essential health services.* A description of each high-impact area is provided below.

#### Community partnerships and engagement

Engaging the community involved builds trust, empowers people, and creates lasting solutions that truly meet the needs of the community. When tackling disease outbreaks, it is essential to include community members, particularly those from marginalized groups [40]. Listening to the insights of community members and collaborating with stakeholders is critical for reaching a wider audience and creating effective containment strategies [41]. Moreover, maintaining open lines of communication with the community can help shape approaches that are more likely to gain support and encourage adherence.

#### Communication

Messages should provide accurate and relevant information about the disease, including risk and protective factors [42]. Information must be culturally sensitive and should avoid labelling. Materials should be linguistically accessible, ensuring that those who do not speak the dominant language or may have visual or auditory disabilities are not excluded. Actively involving community members in all phases of risk communication strategies can lead to successful outcomes. Importantly, messages should be regularly updated as new information is learned [43]. Engaging local stakeholders can also help communities adjust and adapt to these messages as needed.

#### Social and economic conditions

Socioeconomically disadvantaged individuals may face challenges in adhering to response strategies, not because they underestimate the risk but because their immediate survival needs take precedence [44]. For example, people in industries that use a points system for attendance might hesitate to take time off for vaccinations or to recover from being sick due to the risk of accumulating points that could lead to termination. Similarly, those in multigenerational housing may be unable to practice physical distancing [23]. It is crucial to ensure that vulnerable individuals are not stigmatized for not following guidelines, as this is essential for promoting fairness in responses to outbreaks.

#### Data systems and methods

The control of outbreaks depends on the availability of accurate and timely data. Data highlights disparities and can inform the development of interventions. Several factors should be considered. First, partnerships between health agencies and community networks should be reinforced to facilitate timely data sharing [45]. Data generation, storage, and dissemination during health emergencies are necessary for scientific progress. Systems must be established to manage data collection and dissemination openly and transparently while protecting privacy [45]. Data sharing should be approached with an equity lens and a human rights approach [46], and establishing standards to protect patients and research participants is vital [40]. For instance, people and communities ought to understand how their data is utilized, have access to the research results derived from their data, and benefit from those results. Second, to get a nuanced understanding of health inequities, collecting disaggregated data on social factors (e.g., age, race, ethnicity, gender, sexual orientation, citizenship status, geographic factors, etc.) is of utmost importance [47]. However, attempts to address equity concerns may be undermined by policies aimed at specific groups (e.g., LGBTQ+) that can affect participation in data collection and their capacity to share accurate information. Third, research studies are essential sources of data in the context of an outbreak. For instance, serological studies can identify exposure status and ways to create mitigating measures for affected populations; health behavior studies provide evidence of the barriers to response. Likewise, clinical trials can examine new ways to detect or diagnose a disease and test the efficacy of new drugs or vaccines. However, research studies tend to overrepresent individuals from high-income nations and those identifying as white or Caucasian, which limits the applicability of the results [48–50]. Fourth, it is vital to promote the use of both qualitative and quantitative data collection methods. Incorporating anthropological or ethnographic approaches can also strengthen decision-making processes during these emergencies [51].

#### Health infrastructure and supply chains for preventives, therapeutics, and diagnostics

Given that production capacities vary across countries, developing these tools requires a global collaborative effort [52, 53]. Emphasis should be placed on expanding biotechnology by investing in and promoting tools developed locally. For instance, during the early phase of the COVID-19 pandemic, researchers at DiaTropix in Dakar, Senegal, locally manufactured affordable Rapid Diagnostic Tests to improve testing capacity [54]. Additionally, companies owned and operated by individuals of similar cultural identity as the intended populations should be encouraged to create these tools. For example, a Saudi- and Malaysia-based pharmaceutical company is creating halal vaccines to address vaccine hesitancy stemming from religious beliefs [55]. In addition, having sustainable systems for procuring, allocating, and delivering countermeasures and strengthening laboratory capacities are essential for early detection and rapid response.

#### Accessibility of outbreak resources and general health services

Access to essential resources like vaccines, tests, and treatments is crucial for effectively managing outbreaks. It is important to ensure these resources are distributed fairly and that there are enough healthcare workers to meet the needs of the population. Language and technology barriers should be addressed to facilitate the uptake of resources [56, 57]. Furthermore, resources should be affordable. Finally, other health service utilization may decrease during periods of disease emergency. Therefore, maintaining regular health services for other conditions is vital, particularly for those with chronic conditions that require ongoing care.

### Key actions, assessment indicators, and post-response evaluation tools

The framework outlines six high-impact areas and aligns them with key actions. These key actions are overarching objectives that simplify the impact areas into specific, focused actions, ensuring that users understand how to address each impact area in an equitable manner (Table 1). They are designed to target social, economic, structural, and logistical factors, with the goal of ensuring that all populations, particularly marginalized and vulnerable groups, have access to the resources and opportunities they need during these events.

The key actions for community partnerships and engagement improve equity by actively involving local stakeholders to identify and address unique barriers. By leveraging local resources and fostering trust, interventions become culturally appropriate, inclusive, and reflective of community priorities. This collaborative approach not only increases the likelihood of community members utilizing public health measures but also reduces disparities in access and outcomes.

Similarly, for the communication impact area, actions such as creating culturally sensitive and stigma-free messaging, adapting materials for diverse audiences, and monitoring engagement ensure public health information is accessible to everyone. These efforts overcome language and literacy barriers, combat misinformation, and prevent marginalization that can disproportionately harm minority groups. For instance, during the COVID-19 pandemic, the use of racially or ethnically targeted terms in some public dialogue significantly fueled overt violence and bias against individuals of Asian descent, highlighting the critical need for inclusive and culturally sensitive communication strategies [58].

To mitigate social and economic vulnerabilities, the framework advocates for providing safe shelter, strengthening economic security, and promoting social support services. These actions address inequities faced by populations experiencing housing instability, overcrowded living conditions, or financial insecurity. By reducing these vulnerabilities, the framework minimizes the risk of infection and enhances access to necessities, such as food and essential health services, during crises. Furthermore, strengthening health infrastructure and supply chains reduces disparities by ensuring equitable allocation and delivery of vaccines, diagnostics, and therapeutics, especially in underserved areas, and improving local laboratory capacities further ensures timely access to diagnostic services. Finally, actions to improve the accessibility of outbreak resources address logistical barriers, community health worker training, and the continuity of essential services, ensuring that underserved and vulnerable populations can access life-saving interventions.

Each key action is linked with one or more assessment indicators, primarily targeting response efforts during the planning and implementation stages (Supplementary Material). These indicators, framed as questions to guide the assessment process, help users effectively address the key actions. Individuals and organizations can utilize the indicators to shape their response initiatives, with the flexibility to adapt and modify them as needed to align with their specific context and circumstances. Additionally, EER fosters reflection by providing evaluation questions after the response. Users can highlight the successes in their response efforts and identify areas for improvement. This thorough approach promotes fairness and social responsibility in public health efforts during public health emergencies.

### Integration of feedback from community collaborators

Engagement with community collaborators yielded insights that were instrumental in enhancing the EER framework. Community collaborators’ input led to the revision of our action areas and the addition of several assessment indicators. Collaborators shared their experiences during the COVID-19 pandemic and its impact on the populations they serve. (All three collaborators work in Iowa in the Midwestern United States.) Community collaborators also provided feedback about all six high-impact areas: Specifically, they emphasized the importance of actively engaging communities when responding to public health emergencies and highlighted the need for open communication with key stakeholders through regular check-ins to ensure appropriate measures are implemented. Trust was identified as a critical component in developing these partnerships, and the role of community leaders and organizations as intermediaries was recognized as essential in building trust and facilitating early uptake of response measures.

Collaborators reported that, during the COVID-19 pandemic, lack of translated health information and tailored messaging led to delays in access to crucial information for some community members and that this influenced risk perceptions and willingness to get vaccinated or tested for SARS-CoV-2, especially in rural areas. The need for equitable data collection that actively includes information about racial, cultural, and other sociodemographic characteristics was emphasized. Such data could help community leaders ensure that assistance reaches those who need it most and that response efforts can be effectively tailored to local communities. Logistical challenges in accessing preventive measures, such as vaccines, were also highlighted, particularly concerning the maintenance of the cold chain for vaccine storage and transportation in reaching some populations, such as migrant agricultural workers. Meanwhile, access to mental health services was limited, which left vulnerable groups, particularly adolescents, without the support they needed. Many of these young people lived in areas with limited youth shelters and inpatient care options. Finally, collaborators also highlighted the pandemic’s impact on access to health-related social needs (e.g., food, shelter, medication), especially for community members with chronic medical or mental health conditions. We further outline the key points from sessions with community collaborators in Supplementary Material.

### Application of the EER framework

**Fig. 3:**
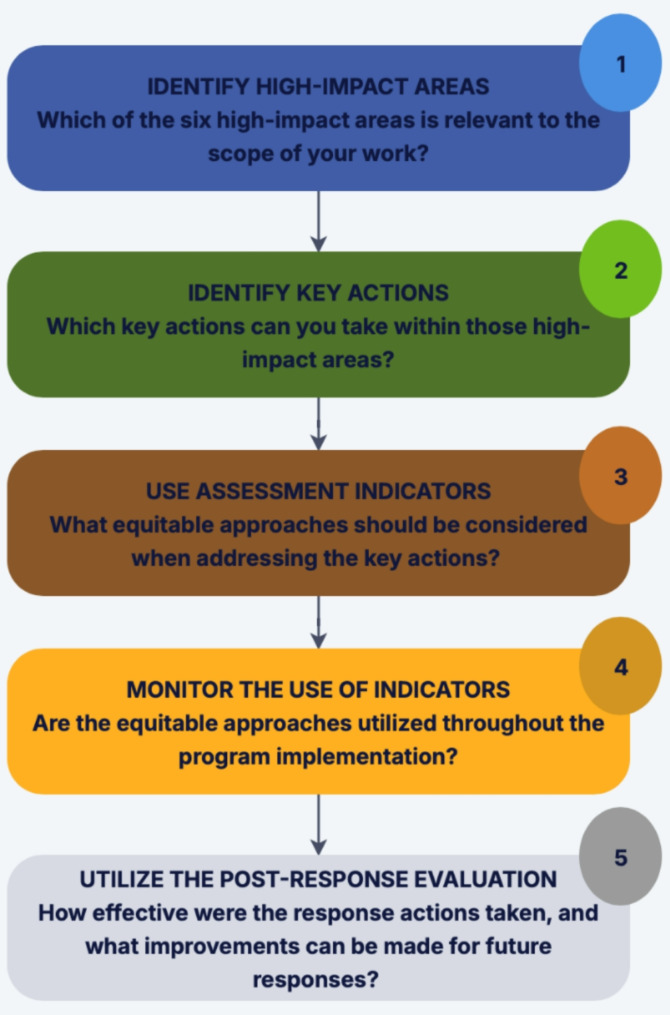
Step-by-step overview for applying the Equity in Epidemic Response framework

The EER framework is designed for public health professionals focusing on epidemic response, global health security, and outbreak surveillance to use the framework as a tool to identify the impact areas that are most relevant to their work and then apply and assess the associated action strategies. The framework is also intended to help public health researchers set research objectives and create relevant questions for exploring epidemic responses. Figure 3 gives an overview of how to use the EER framework. Users can start by identifying the impact area most relevant to their work such as data systems and methods or communication. Next, users can identify key actions within those areas that advance equity—for instance, “engaging community stakeholders in designing and disseminating communication plans and materials.” The assessment indicators can then be used to inform more equitable and effective planning and execution stages of the response. Users are encouraged to consistently track these indicators throughout the implementation of their programs to confirm that equitable methods are being applied and to adjust these efforts as necessary. For instance, stakeholders should be engaged throughout the entire response process, not only at the start. Finally, users can complete the post-response evaluation provided with the framework to assess the response actions taken and identify lessons learned and areas for improvement to enhance future responses.

## Discussion

The health and social consequences of the COVID-19 pandemic across populations worldwide have underscored the critical need for incorporating an equity-focused approach in pandemic and epidemic responses. The evidence is replete with examples of this: For instance, in several underserved communities in Massachusetts, barriers to SARS-CoV-2 testing included limited accessibility of testing sites and long wait times [4]. Meanwhile, in Austria, vulnerable populations, such as older adults and those from economically disadvantaged backgrounds, struggled with accessing pandemic-related health information via the internet due to the digital divide [20]. Likewise, disparities in vaccine distribution and access globally were striking: High-income countries’ early ownership of more than half of the global vaccine doses purchased, despite representing only a fifth of the global adult population, substantially limited the availability of doses for low- and middle-income countries [59, 60]. Community collaborators involved in the development of this EER framework also reported barriers to effective and equitable pandemic responses in community settings, citing barriers faced by people from rural areas, people from medically underserved populations, and people with disabilities or chronic medical or mental health conditions—as well as barriers faced by community organizations and providers who serve these populations. As a result, people with limited pre-pandemic resources faced further depletion of these resources during the pandemic (e.g., employment, housing, food), thereby impacting their short-term health during the pandemic and their long-term health and well-being for years to come. These compounding impacts of public health emergencies on vulnerable populations reinforce the need for a framework like EER, which considers social and structural determinants of health as fundamental aspects of a successful pandemic/epidemic response strategy to minimize morbidity and mortality.

EER is intended to advance epidemic equity by promoting fairness in multiple dimensions of the pandemic or epidemic response. It emphasizes collaborative efforts that are inclusive and culturally appropriate, aims to enhance social and economic conditions that fuel many pandemic/epidemic health risks, ensures ethical and comprehensive data collection and sharing, boosts laboratory capabilities and the creation of epidemic resources, and strives to make health resources more available to everyone.

Designed as a practical guide, EER supports public health professionals in designing and implementing equity-focused response actions. Its flexibility allows indicators to be modified to address the unique social, economic, and cultural needs of different settings while ensuring that the framework’s core principles of equity and inclusivity remain actionable and relevant. By providing a structured yet flexible approach, the EER framework allows users to tailor response actions effectively while maintaining a foundational focus on health equity.

### Limitations

This work has some important limitations. First, the EER framework does not directly address the critical, practical role of funding. Public health organizations need sufficient financial resources to execute strategies successfully. Likewise, while we highlight the value of supporting locally developed resources (e.g., testing assays, biologics) wherever possible, we recognize that scientific challenges and funding limitations may also pose barriers to these efforts in some settings. Third, despite the purposeful inclusion of collaborators with diverse expertise in EER framework development, most collaborators’ lived experiences are from the global north. Thus, the framework’s applicability in other settings may be limited. Although the framework was developed with a relatively small group of experts, it is intended as a model for adaptation in other settings by iteratively engaging stakeholders across the sectors outlined in the framework in the context of specific public health needs. Finally, measuring and evaluating the effectiveness of equity-based interventions during rapidly evolving emergencies presents inherent challenges due to the dynamic nature of such situations. However, the framework provides a useful tool for systematically documenting actions and tracking progress. Users can track the indicators they initially selected throughout the program’s implementation to ensure that equitable approaches are consistently applied. This structured monitoring process enables users to evaluate the alignment of their actions with equity goals, both during and after response efforts, allowing them to assess impact and identify opportunities for improvement.

## Conclusion

The EER framework provides a comprehensive approach to a pandemic/epidemic response. Focusing on the action areas and indicators proposed in this framework can advance public health efforts to combat an epidemic’s short-term and long-term impacts on public health and promote health equity. Our framework is enhanced by partnerships with collaborators from academic and community settings. However, the framework’s features are dynamic; these can and should continue to evolve over time and in response to emerging public health crises with iterative input from stakeholders who can guide progress toward each action area and indicator in realtime. We intend to expand on this work by collaborating with health professionals whose expertise was not represented in the initial development of this work, including those in anthropology, humanities, pharmacy, and more, to ensure that the framework remains pertinent and practical for its intended audience. We also plan to qualitatively assess the relevance of this work among target users.

## Electronic supplementary material

Below is the link to the electronic supplementary material.

Supplementary Material 1

## Data Availability

Data sharing is not applicable to this article as no datasets were generated or analyzed during the current study.

## Abbreviations

COVID-19: Coronavirus disease 2019
SARS-CoV-2: Severe acute respiratory syndrome coronavirus 2
EER: Equity in Epidemic Response
IHJ-JEE: International Health Regulations- Joint Evaluation Tool
GHSI: Global Health Security Index
PIP: Pandemic Influenza Plan
HiAP: Health in All Policies Framework
