# Supplementary material for: Equity in epidemic response: an action-oriented framework for guiding public health in equitable responses to major infectious disease emergencies
Source: Int J Equity Health. 2025 Mar 12;24:69. doi: 10.1186/s12939-025-02433-2 (PMC11900141; doi:10.1186/s12939-025-02433-2)
Supplement: Supplementary file 1 — Supplementary Material 1 [file 12939_2025_2433_MOESM1_ESM.docx]

**Equity in Epidemic Response: An Action-Oriented Framework for Guiding Public Health in Equitable Responses to Major Infectious Disease Emergencies (de-Winton Cummings, et al.)**

**Supplementary Material**

**Feedback from Community Partners Mapped to the Six High-Impact Areas**

| **High-Impact Area** | **Feedback from non-academic collaborators/community partners** | **Some examples of indicators informed by these sessions** |
| --- | --- | --- |
| **Community Partnerships and Engagement** | Our community partners stressed the importance of engagement and partnerships in responding to disease threats. Regular meetings and check-ins among key stakeholders are important in ensuring that appropriate measures are being implemented. For example, organizations serving agricultural workers had daily check-ins with the local and state government, employers, laboratories and healthcare personnel to discuss strategies, share information, and facilitate testing for new employees. Trust was also highlighted as crucial within the context of engagement and partnerships. Community leaders and organizations were recognized as essential in building trust with the community and could potentially act as intermediaries between the community and service providers to allow for early uptake of response measures.    There was a consensus among collaborators that researchers and practitioners should spend time in communities to understand where they are coming from and the challenges they face. It was also emphasized that the perspectives of vulnerable populations should be directly considered, as they are best positioned to express their needs, and as such, their voices should be heard and prioritized in decision-making processes. | Are local organizations (*e.g. faith-based organizations, non-profits etc.*) or local healthcare providers engaged in the planning and implementation of response measures? |
| **Communication** | In culturally diverse communities, health information materials were predominantly in English. Translating these materials took a long time due to a lack of personnel and translation services. Consequently, these communities lacked timely access to current information, as new updates were available by the time translations were finished. In addition to language barriers, the content of the messages was also limited, lacking targeted messaging. For example, migrant workers from countries with lower disease burden had a low-risk perception about the pandemic. However, messaging to this population prioritized information about identifying symptoms, social distancing, etc., without promoting positive attitudes and perceptions regarding the disease. This may have contributed to a lower intention to vaccinate or test for SARS-CoV-2 infection, especially in rural areas where risk perceptions were lower. Collaborators also highlighted the limited availability interpreters in hospital settings can deter non-English speakers from utilizing services during disease emergencies. | Are messages provided in multiple languages that are reflective of the diversity in the population?  Are the perceptions, beliefs and concerns of individuals receiving these messages being monitored? |
| **Data Systems and Methods** | The main considerations regarding this impact area revolve around evaluating the effectiveness of response strategies. Collaborators stressed the crucial nature of thoroughly assessing the individuals receiving services to ensure that those with the most pressing needs are reached. This involves identifying specific points of access for proactive measures like vaccines and closely monitoring the demographic profile of vaccine recipients. For instance, tracking the number of distributed vaccines, amount of distributed food, user demographics, service locations, and accessibility.    Embracing a health equity approach is of utmost importance to gather and disseminate detailed data on racial, cultural, and socio-demographic characteristics. One collaborator pointed out the need for the use of multidisciplinary research and data collection methods. | Does the research use an interdisciplinary approach, such as collecting data through ethnographic, anthropological, or other methods? |
| **Health Infrastructure and Supply Chains for Preventives, Therapeutics, and Diagnostics** | Access to vaccines was facilitated through the use of mobile clinics. Notwithstanding their effectiveness, logistical challenges arose, particularly in maintaining the cold chain for vaccine storage and transportation. Consequently, some populations, such as migrant workers, only had access to the Moderna and Johnson & Johnson vaccines through mobile clinics due to this constraint. | Are there any existing structures or established institutions in low-resource settings or hard-to-reach areas that can facilitate the rapid development and distribution of vaccines, including the availability of cold storage? |
| **Accessibility of Outbreak Resources and Essential Health Services** | There were differences in accessibility to outbreak-related resources such as vaccines and tests across vulnerable groups. For instance, migrant workers had access to testing through their employers, while other vulnerable populations, particularly those in rural areas, faced challenges in accessing the statewide testing program due to physical inaccessibility and limited accommodation factors such as computer and internet barriers to schedule appointments. Effective contact tracing of migrant workers was challenging as many workers were unaware of their close contacts during their communal commute to the United States.    One collaborator stressed the importance of providing culturally inclusive health services/culturally competent care so that individuals from marginalized populations can feel comfortable utilizing those services.    Access to other essential health services, such as mental health services, was also constrained. The impact of disease emergencies, such as the COVID-19 pandemic, indirectly affects adolescents and youths. School closures and the disruptions caused by social distancing measures have significantly affected the mental and social well-being of this group, potentially leading to risky behaviors like substance abuse or self-harm. Immigrant adolescents, in particular, are at heightened risk due to the challenges of adapting to a new system and culture, exacerbating the pandemic's impact on their mental health. In rural areas where there is a high proportion of culturally diverse immigrant populations, there is a scarcity of youth shelters or in-patient services, and the average wait time for services is three months, exacerbating the conditions for these adolescents. | Are there functioning patient-centered services for people with substance use disorders to provide harm reduction services during disease emergencies? |
| **Social and Economic Conditions** | Access to basic necessities such as food, shelter, and medication is absolutely vital, especially for individuals with mental and thought disorders who rely on these critical services. Disruptions in health services during the pandemic, along with limited funding for organizations working with these populations, made it increasingly difficult for individuals to obtain essential resources. It became the sole responsibility of these organizations/groups to provide these resources. This was particularly pronounced in rural areas, where access to healthcare and basic amenities is already constrained. As a result, many individuals grappling with these disorders have been forced into homelessness and are facing significant food insecurity, further compounding the challenges they face due to their conditions.    One collaborator stressed the importance of building relationships with /and providing funding for local shelters, crisis centers and food banks to ensure that they are equipped to provide resources to those in need. Another collaborator emphasized the importance of providing strict protection for workers. They highlighted the dilemma faced by economically disadvantaged individuals in certain industries who had to risk exposing themselves and others in order to provide for their families. Similarly, the importance of sufficient staffing, pay equity, financial reimbursements, and time off for frontline workers, including community health workers, who may be overworked during emergencies was highlighted. | Is it possible to connect individuals facing homelessness  to affordable, safe, sanitary, and secure short-term housing? (*examples of short-term housing include shelters, extended stay motels, hotels etc.*)  Are there restrictions for eligibility?  Is the safety of health workers, including community health workers, being monitored, and addressed, such as providing support for stress and burnout, paid time off, and access to PPEs during disease emergencies? |
| Collaborators also shared some important recommendations that were not mapped to the six-high impact areas or that applied across multiple areas. Below are some key points:   - Avoid saviorism in health/ healthcare. - Increase the availability of providers with a similar cultural identity to the community they are serving. Additionally, individuals working in communities different from their own should possess basic knowledge about the cultures they are providing care for. - Address the challenges with contact tracing for specific groups, such as migrant workers. - Acknowledge the human element in scientific research and ensure that researchers prioritize the immediate needs of communities while collecting data. - Provide public health training to medical providers so they can understand the rationale behind response efforts and effectively contribute to public health initiatives. | | |

**Guide for Using the Assessment Indicator Tools for Each Domain**

This tool's primary function is to guide the development and implementation of response strategies. This guide outlines the steps necessary to effectively employ the assessment indicator tables for each domain, ensuring a comprehensive and equitable response during public health emergencies.

1. **Designate the Stage of Response Efforts**

To begin using the assessment indicator tools, users must first identify the stage of their response efforts. This includes:

- Response Planning: this stage involves formulating strategies and actions to address the disease emergency.
- Response Implementation: this stage comprises executing planned strategies during the disease emergency.

1. **Identify Key actions**

After designating the stage of the response effort, users should identify the key action areas most pertinent to their roles or interests. This helps focus the assessment on the most relevant aspects of the response.

1. **Utilize Assessment Indicators and Monitor Progress**

For each key action area, users will use assessment indicators to evaluate and guide their efforts. The process differs slightly depending on the response stage:

**During the Response Planning Stage**:

- **Examine Indicators**: Users should review the indicators to identify essential considerations as they develop their strategies.
- **Select Progress Indicators**: For each assessment indicator considered, users should denote whether it has been considered by selecting "yes,” "no,” or " In progress.”
- **Capture Supplementary Details**: In the notes section, users can record potential challenges addressing the selected indicators and any other relevant information.

**During the Response Implementation Stage**:

- **Select Progress Indicators**: For each indicator, users should indicate the progress by selecting "yes,” "no,” or "In progress” to denote whether it was utilized in the response effort
- **Capture Supplementary Details**: In the notes section, users can document challenges encountered, insights gained, and the time and resources invested in addressing the indicators.

*Note: P= Response Planning Stage; I= Response Implementation Stage

**ASSESSMENT INDICATORS**

***Note: Some aspects of this framework refer to vulnerable groups in general. We consider vulnerable groups to include, but not be limited to, older adults, pregnant women, children, racial and ethnic minority groups, low-income individuals, people with substance use disorders, people living with HIV, and people living with a disability. We urge users to adapt these indicators based on the vulnerable groups they serve.***

**Community Partnerships and Engagement**

| **Stage** | | **Key Actions** | **Indicators** | **Progress** | | | | | |
| --- | --- | --- | --- | --- | --- | --- | --- | --- | --- |
| **Response Planning** | **Response Implementation** |  |  | **No** | **Yes** | | **In Progress (if applicable)** | | **Notes** |
|  |  |  |  |  | **P** | **I** | **P** | **I** |  |
|  |  | **Engage with community stakeholders to identify needs and address barriers with response strategies~~.~~** | Are community-elected representatives from under-served communities engaged in the planning and implementation of response measures during disease emergencies? |  |  |  |  |  |  |
|  |  |  | Are local organizations (*e.g. faith-based organizations, non-profits etc.*) or local healthcare providers engaged in the planning and implementation of response measures during disease emergencies? |  |  |  |  |  |  |
|  |  |  | Have the needs and barriers to public health measures during disease emergencies been directly identified by community members? |  |  |  |  |  |  |
|  |  |  | Is there a bidirectional flow of information with communities during disease emergencies? |  |  |  |  |  |  |
|  |  |  | Is community input and participation documented? |  |  |  |  |  |  |
|  |  | **Implement interventions utilizing local resources~~.~~** | Are community resources being utilized to ensure that large proportions of the population can be reached or have access to resources  during disease emergencies (*e.g., use of community health workers for surveillance, use of parking lots for vaccination, etc.*)? |  |  |  |  |  |  |
|  |  | **Practice cultural humility and prioritize building trust in communities~~.~~** | Have  there been cultural competency and cultural humility trainings for individuals that will be working with specific communities during disease emergencies? |  |  |  |  |  |  |
|  |  |  | Have there been other trainings to ensure that individuals are knowledgeable about the communities that they will be working in during disease emergencies? |  |  |  |  |  |  |
|  |  |  | Are strategies being implemented  to actively build trust in communities during disease emergencies to ensure adherence and uptake of measures? |  |  |  |  |  |  |

**Communication**

| **Stage** | | **Key Actions** | **Indicators** | **Progress** | | | | | | | | | |
| --- | --- | --- | --- | --- | --- | --- | --- | --- | --- | --- | --- | --- | --- |
| **Response**  **Planning** | **Response Implementation** |  |  | **No** | **Yes** | | | **In Progress**  **(if applicable)** | | | | | **Notes** |
|  |  |  |  |  | **P** | | **I** | **P** | | | | **I** |  |
|  |  | **Engage community stakeholders in designing and disseminating communication plans and materials.** | Are there regular meetings or daily check-ins between stakeholders and response agencies, including local and state departments to facilitate the dissemination of health information during these emergencies?  *Make a note of how often these meetings are held. |  |  |  | |  | | | |  |  |
|  |  |  | Are messages thoroughly evaluated by community representatives during disease emergencies for accuracy and cross-cultural effectiveness before dissemination? |  |  |  | |  | | | |  |  |
|  |  | **Develop culturally aware messages to prevent stigma, labeling or othering of populations.** | Are the messages developed during disease emergencies constructed in ways that are culturally aware and respectful to all individuals and communities? |  |  |  | |  | | | |  |  |
|  |  |  | Have there been trainings on developing culturally aware materials in collaboration with communities? |  |  |  | |  | | | |  |  |
|  |  |  | Do the design and implementation of communication strategies consider the tone of the message (*e.g., are the messages created to avoid language that may incite fear or perpetuate stigma*)? |  |  |  | |  | | | |  |  |
|  |  | **Adapt messages so that information is readily accessible and comprehensible.** | Are messages regularly adapted to meet the needs and concerns of different populations? |  |  |  | |  | |  | | |  |
|  |  |  | Are messages provided in multiple languages that are reflective of the diversity in the population? |  |  |  | |  | |  | | |  |
|  |  |  | Are informational materials accessible to individuals with learning, reading, or vision-related disabilities? |  |  |  | |  | |  | | |  |
|  |  |  | Are messages designed in layman’s terms to ensure that anyone can understand regardless of educational level (*e.g. using appropriate and culturally sensitive images*)? |  |  | |  | |  | |  | |  |
|  |  | **Monitor engagement with health messages.** | Are the perceptions, beliefs, and concerns of individuals receiving these messages being monitored? |  |  | |  | |  | |  | |  |
|  |  |  | Is the content of messages regularly monitored to identify discriminatory and stigmatizing language(s)? |  |  | |  | |  | |  | |  |
|  |  |  | Are communities regularly engaged to identify their interactions, concerns and questions about health messages? |  |  | |  | |  | |  | |  |
|  |  |  | Are there mechanisms to regularly monitor the frequency of disseminated messages, types of information media utilized, and whether the messages reach the targeted audience? |  |  | |  | |  | |  | |  |
|  |  |  | If possible: At the local or state level, are infodemiology (social media surveillance) studies conducted to analyze social media content and to identify priorities in addressing health misinformation? |  |  | |  | |  | |  | |  |

**Social and Economic Conditions**

| **Stage** | | **Key Actions** | **Indicators** | **Progress** | | | | | |
| --- | --- | --- | --- | --- | --- | --- | --- | --- | --- |
| **Response Planning** | **Response Implementation** |  |  | **No** | **Yes** | | **In progress**  **(if applicable)** | | **Notes** |
|  |  |  |  |  | **P** | **I** | **P** | **I** |  |
|  |  | **Provide safe and temporary shelter.** | Are there ways to connect people to affordable, safe, sanitary, and secure short-term housing (e.g., *shelters, extended stay motels, hotels etc.*)? |  |  |  |  |  |  |
|  |  |  | If these resources available, are they accessible to people who have housing instability or insecurity due to socioeconomic factors, medical needs, disability, or concerns about violence/safety?  *Note if there are restrictions for eligibility. |  |  |  |  |  |  |
|  |  |  | Can high-risk individuals who may not be able to protect themselves at home due to factors such as unstable housing or overcrowding be connected to affordable, safe, sanitary, and secure short-term housing options?  *Note if there restrictions for eligibility (*e.g. people at greater risk of infection due to host factors or occupation).* |  |  |  |  |  |  |
|  |  | **Strengthen economic security.** | Are there relief programs that can provide financial assistance to at-risk individuals and small businesses during disease emergencies? |  |  |  |  |  |  |
|  |  |  | If relief programs are available, are they monitored to determine who has access to them? |  |  |  |  |  |  |
|  |  |  | Are there programs or organizations that advocate for essential occupations to ensure employers provide benefits like paid sick time and flexible hours without any negative consequences during disease emergencies (e.g. being terminated)? |  |  |  |  |  |  |
|  |  | **Promote social support services.** | Are there any efforts being made to reduce the stigma surrounding mental health and increase the use of mental health services during disease emergencies? |  |  |  |  |  |  |
|  |  |  | Are services available to provide individuals or families with timely and affordable mental health and psychosocial support during disease emergencies? |  |  |  |  |  |  |
|  |  |  | Are mental health programs available to vulnerable groups including adolescents and children, individuals with substance use disorders, nonimmigrants/ immigrants etc. during disease emergencies? |  |  |  |  |  |  |
|  |  |  | Are culturally aware approaches integrated into counselling or mental health care provided during this period? |  |  |  |  |  |  |
|  |  |  | Are community stakeholders, such as religious leaders, leaders of minority groups etc., being utilized to provide psychosocial (such as *emotional, spiritual, social*) support to their respective communities during disease emergencies? |  |  |  |  |  |  |

**Data Systems and Methods**

| **Stage** | | **Key Actions** | **Indicators** | **Progress** | | | | | |
| --- | --- | --- | --- | --- | --- | --- | --- | --- | --- |
| **Response Planning** | **Response Implementation** |  |  | **No** | **Yes** | | **In Progress (if applicable)** | | **Notes** |
|  |  |  |  |  | **P** | **I** | **P** | **I** |  |
|  |  | **Establish mechanisms for equitable and ethical data collection and sharing with researchers and communities.** | Are there training sessions for data collection methods during disease emergencies? |  |  |  |  |  |  |
|  |  |  | Are community members actively involved in assisting with data collection during disease emergencies? |  |  |  |  |  |  |
|  |  |  | Is there an efficient and timely system in place to enable ethical sharing of data with healthcare practitioners and researchers during disease emergencies? |  |  |  |  |  |  |
|  |  |  | Are there privacy policies that explain how collected personal information will be handled? |  |  |  |  |  |  |
|  |  |  | Are there security measures to protect personal data, including the storage and sharing of data? |  |  |  |  |  |  |
|  |  | **Increase representation of marginalized populations in research studies.** | Are there any strategies to ensure that underrepresented populations are well-represented in research studies during disease emergencies (*e.g. building trust, practicing cultural humility, acknowledging power imbalances and historical trauma)?* |  |  |  |  |  |  |
|  |  |  | Are community members included in ethical review committees for research studies during disease emergencies to ensure that the research takes into account the needs and concerns of the community, ultimately leading to more ethical and effective outcomes? |  |  |  |  |  |  |
|  |  | **Implement or strengthen community-based surveillance.** | Are members of the community involved in community-based surveillance measures, such as contact tracing or reporting of cases during disease emergencies? |  |  |  |  |  |  |
|  |  |  | Are there training programs available that would enable community members to conduct community-based surveillance? |  |  |  |  |  |  |
|  |  |  | Is there any provision of funds to compensate community members who perform surveillance duties during disease emergencies? |  |  |  |  |  |  |
|  |  |  | Is there a community health worker network or workforce that can perform surveillance duties during disease emergencies? |  |  |  |  |  |  |
|  |  | **Utilize qualitative and quantitative methods to design and analyze research studies.** | Are community members or partners included in the development of research objectives, validation of measures or instruments, recruitment etc? |  |  |  |  |  |  |
|  |  |  | Does the research use an interdisciplinary approach, such as collecting data through ethnographic, anthropological, or other methods? |  |  |  |  |  |  |
|  |  |  | Is data being utilized to identify barriers to resources during disease emergencies? |  |  |  |  |  |  |
|  |  |  | Is data being utilized to determine effective resource allocation strategies during disease emergencies? |  |  |  |  |  |  |
|  |  |  | Are study findings, including preliminary results, being shared at no cost and in a timely manner with communities during disease emergencies? |  |  |  |  |  |  |
|  |  |  | Are the results of studies presented in a way that is easy to understand? |  |  |  |  |  |  |

**Health Infrastructure and Supply Chains for Preventives, Therapeutics and Diagnostics**

| **Stage** | | **Key Actions** | **Indicators** | **Progress** | | | | | | | | | | | | | |
| --- | --- | --- | --- | --- | --- | --- | --- | --- | --- | --- | --- | --- | --- | --- | --- | --- | --- |
| **Response Planning** | **Response Implementation** |  |  | **No** | **Yes** | | | | **In Progress**  **(if applicable)** | | | | | | | | **Notes** |
|  |  |  |  |  | **P** | | **I** | | | | **P** | | **I** | | | |  |
|  |  | **Promote partnerships to expand the production of preventives, therapeutics, and diagnostics.** | Are there partnerships with local biotech companies to increase the production of preventives, therapeutics, and diagnostics during disease emergencies? |  | |  | | |  | |  | |  | | |  | |
|  |  |  | Are there partnerships with culturally appropriate companies or institutes to anticipate, identify and respond to cultural or religious needs regarding the production of preventives, therapeutics, and diagnostics during disease emergencies? |  | |  | | |  | |  | |  | | |  | |
|  |  |  | Are preventives, therapeutics, and diagnostics developed locally promoted for global use during disease emergencies? |  | |  | | |  | |  | |  | | |  | |
|  |  | **Develop an equitable mechanism for procuring, allocating, and delivering vaccines, therapeutics, and diagnostics.** | Are representatives from historically marginalized groups, including minority racial groups, low- and middle-income countries, etc. included in the decision-making process of resource development and distribution during disease emergencies? |  | |  | | |  |  | | |  |  | | | |
|  |  |  | Are there any existing structures or established institutions in low-resource settings or hard-to-reach areas that can facilitate the rapid development and distribution of vaccines during disease emergencies, including the availability of cold storage? |  |  | | |  | | |  |  | | |  | | |
|  |  |  | Is there a simple and accessible open-access resource that tracks the development and distribution process? |  |  | | |  | | |  |  | | |  | | |
|  |  | **Strengthen local laboratory capacities.** | Are local certified laboratory services, such as for infection testing, available to the public during disease emergencies? |  |  | | |  | | |  |  | | |  | | |
|  |  |  | Is the utilization of laboratory services monitored to identify who has access to them during disease emergencies? |  |  | | |  | | |  |  | | |  | | |
|  |  |  | Are laboratory services affordable for the entire population during disease emergencies? |  |  | | |  | | |  |  | | |  | | |
|  |  |  | Can the laboratory provide timely diagnosis during disease emergencies? **Note the average turn-around time for test results.* |  |  | | |  | | |  |  | | |  | | |
|  |  |  | Does the laboratory have all the necessary resources and equipment for large-scale testing during disease emergencies? |  |  | | |  | | |  |  | | |  | | |
|  |  |  | Can the laboratory manage the workload during disease emergencies with its current staff, or will it need additional human resources? |  |  | | |  | | |  |  | | |  | | |
|  |  |  | Does laboratory data reporting during disease emergencies include information on socio-demographics and health-related social needs?  **Note the types of factors that are collected.* |  |  | | |  | | |  |  | | |  | | |
|  |  |  | Is essential PPE accessible to laboratory staff during disease emergencies? |  |  | | |  | | |  |  | | |  | | |

**Accessibility of Outbreak Resources and Essential Health Services**

| **Stage** | | **Key Actions** | **Indicators** | **Progress** | | | | | | | | | |  |  |  |  |  |
| --- | --- | --- | --- | --- | --- | --- | --- | --- | --- | --- | --- | --- | --- | --- | --- | --- | --- | --- |
| **Response Planning** | **Response Implementation** |  |  | **No** | **Yes** | | | | **In Progress**  **(if applicable)** | | | **Notes** | |  |  |  |  |  |
|  |  |  |  |  | **P** | | **I** | | **P** | | **I** |  |  |  |  |  |  |  |
|  |  | **Address barriers that may influence the uptake of public health measures.** | Are disparities in accessing resources, such as those related to social determinants of health and discrimination, monitored during disease emergencies? |  | |  |  |  | |  | | |  |  |  |  |  |  |
|  |  |  | Are cost, geographic, and other accommodation barriers to getting resources evaluated and addressed during disease emergencies? |  | |  |  |  | |  | | |  |  |  |  |  |  |
|  |  | **Develop sustainable supply and distribution strategies.** | Are there partnerships with local stakeholders such as pharmacies, clinics/hospitals, and public/private community-based entities, to facilitate sustainable supply and distribution of resources during disease emergencies? |  | |  |  |  | |  | | |  |  |  |  |  |  |
|  |  |  | Is the distribution and uptake of resources (*e.g., number of tests performed, vaccines distributed, etc.*) monitored during disease emergencies? |  | |  |  |  | |  | | |  |  |  |  |  |  |
|  |  |  | Are resource allocation strategies informed by qualitative or quantitative research studies conducted during the disease emergency? |  | |  |  |  | |  | | |  |  |  |  |  |  |
|  |  |  | Are resource allocation strategies during disease emergencies informed by the needs and priorities of communities reported by the communities themselves? |  | |  |  |  | |  | | |  |  |  |  |  |  |
|  |  | **Provide training and support for health workers, including community health workers.** | Do health workers, including community health workers, receive training to perform their duties effectively during disease emergencies? |  | |  |  |  | |  | | |  |  |  |  |  |  |
|  |  |  | Is the safety of health workers, including community health workers, being monitored, and addressed (i.e., providing support for stress and burnout, paid time off, and access to PPEs during disease emergencies)? |  | |  |  |  | |  | | |  |  |  |  |  |  |
|  |  | **Maintain the provision of other essential health services such as care for chronic conditions, substance use disorders, sexually transmitted infections (STIs), etc.** | Are health promotion strategies being utilized during disease emergencies to encourage people with other health conditions such as chronic conditions, substance use disorders, sexually transmitted infections, etc. to seek medical health? |  | |  |  |  | |  | | |  |  |  |  |  |  |
|  |  |  | Are there any effective strategies to ensure that individuals living with chronic conditions adhere to their treatment regimen during disease emergencies? |  | |  |  |  | |  | | |  |  |  |  |  |  |
|  |  |  | In situations where routine monitoring is not feasible, are there alternative methods of delivering healthcare services? For instance, can community health workers or home health aides be utilized for home visits? |  | |  |  |  | |  | | |  |  |  |  |  |  |
|  |  |  | Is there a referral system in place to manage medical conditions that require time-sensitive interventions during disease emergencies? |  | |  |  |  | |  | | |  |  |  |  |  |  |
|  |  |  | If telemedicine options are being utilized, are there mechanisms in place to address technology and digital barriers during disease emergencies? |  | |  |  |  | |  | | |  |  |  |  |  |  |
|  |  |  | Are there functioning patient-centered services for people with substance use disorders to provide harm reduction services during disease emergencies? |  | |  |  |  | |  | | |  |  |  |  |  |  |
|  |  |  | Are routine immunization services maintained during disease emergencies, especially for children and for adults over 65 years of age? |  | |  |  |  | |  | | |  |  |  |  |  |  |
|  |  |  | Are STI testing services available during disease emergencies? Are self-testing options available? |  | |  |  |  | |  | | |  |  |  |  |  |  |

**Guide for Using the Post-Response Evaluation Tool**

**Stage: Post-Response**

The post-response stage encompasses evaluating completed response efforts. While this framework primarily guides users in developing and implementing strategies during disease emergencies, including a post-response stage allows users to evaluate their response efforts critically. This stage encourages a comprehensive reflection on what was planned versus what was executed, identifying successes, challenges, and areas for improvement.

**Purpose**: The post-response evaluation aims to provide a structured approach to assess the effectiveness of response strategies, identify discrepancies, and document lessons learned. This reflective process is essential for continuous improvement and ensuring that future responses are more effective and equitable.

**Responding to the Post-Response Evaluation Tool using the Notes Column**

**Indicator Alignment:** Users are encouraged to compare the indicators selected during the planning stage with those addressed during the implementation stage. This comparison helps to evaluate whether the planned actions were successfully implemented and to what extent the intended outcomes were achieved.

**Challenges:** Users should document any challenges encountered or reasons for discrepancies between the planned actions and actual implementation. This includes logistical issues, resource constraints, unforeseen circumstances, or any other factors that impacted the response efforts.

**Adjustment and Improvements:** Users should capture insights gained, time and resources invested, and any adjustments they would recommend for future strategies.

**Post-Response Evaluation Questions**

| **Post-Response Evaluation Domains** | **Post-Response Evaluation Questions** | **Notes** |
| --- | --- | --- |
| **Indicator Alignment** | Which indicators were selected during the planning stage? |  |
|  | Were the indicators selected during the planning stage addressed during the implementation stage? |  |
|  | Which indicators were successfully achieved as planned, and which ones faced challenges? |  |
| **Challenges** | What are the main challenges or reasons for discrepancies between planned actions and actual implementation of each indicator? |  |
|  | How did these challenges impact the overall effectiveness of response effort? |  |
| **Adjustment and Improvements** | Based on the comparison of planning and implementation indicators, what adjustments or improvements would be recommended for future response efforts? |  |
|  | How can these recommendations enhance the effectiveness of similar response strategies in the future? |  |
